# Supplementary material for: Integrating quantitative and qualitative methodologies to build a national R&D plan using data envelopment analysis based on R&D stakeholders’ perspectives
Source: PLoS One. 2022 Mar 11;17(3):e0265058. doi: 10.1371/journal.pone.0265058 (PMC8916661; doi:10.1371/journal.pone.0265058)
Supplement: S1 Table — (PDF) [file pone.0265058.s001.pdf]

S1 Table. Comparison of efficiency scores and ranks between the DEA and DEA-AR models

DMU	Efficiency Score					Ranking				
	DEA	DEA-AR				DEA	DEA-AR			
		Total	Industries	Academia	R.I		Total	Industries	Academia	R.I
T1	0.167	0.146	0.143	0.145	0.144	11	9	8	9	8
T2	0.040	0.009	0.006	0.008	0.008	18	20	20	20	20
T3	0.261	0.243	0.240	0.243	0.241	8	4	4	4	4
T4	0.264	0.260	0.258	0.260	0.258	7	3	3	3	3
T5	0.071	0.038	0.037	0.038	0.038	16	14	14	14	14
T6	0.255	0.213	0.208	0.212	0.210	9	5	5	5	5
T7	0.503	0.095	0.093	0.095	0.094	5	11	11	11	11
T8	0.025	0.019	0.018	0.019	0.019	20	15	15	15	15
T9	0.198	0.118	0.117	0.118	0.117	10	10	9	10	10
T10	0.617	0.422	0.406	0.420	0.416	3	2	2	2	2
T11	0.597	0.155	0.148	0.154	0.153	4	8	7	7	7
T12	0.017	0.003	0.002	0.003	0.003	21	21	21	21	21
T13	1.000	0.158	0.094	0.148	0.144	1	7	10	8	9
T14	0.270	0.168	0.161	0.167	0.167	6	6	6	6	6
T15	0.112	0.017	0.010	0.016	0.015	13	17	18	17	17
T16	1.000	1.000	1.000	1.000	1.000	1	1	1	1	1
T18	0.062	0.053	0.052	0.053	0.052	17	13	13	13	13
T19	0.103	0.012	0.010	0.012	0.012	14	19	19	19	19
T20	0.008	0.002	0.001	0.002	0.002	23	23	23	23	23
T21	0.003	0.000	0.000	0.000	0.000	24	24	24	24	24
T22	0.013	0.002	0.002	0.002	0.002	22	22	22	22	22
T23	0.025	0.013	0.012	0.013	0.013	19	18	17	18	18
T25	0.096	0.085	0.084	0.085	0.084	15	12	12	12	12
T26	0.115	0.017	0.014	0.017	0.017	12	16	16	16	16

Note: DMUs T17 and T24 are excluded because their input values are zero. R.I represents Research Institutes.
