# Supplementary material for: Integrating quantitative and qualitative methodologies to build a national R&D plan using data envelopment analysis based on R&D stakeholders’ perspectives
Source: PLoS One. 2022 Mar 11;17(3):e0265058. doi: 10.1371/journal.pone.0265058 (PMC8916661; doi:10.1371/journal.pone.0265058)
Supplement: S2 Table — (PDF) [file pone.0265058.s002.pdf]

S2 Table 1. Results of the DEA and DEA-AR model analyses (industries)

DMU	e	ar	L13	L16	sx_1	sy_1	sy_2	sy_3	sy_4	sy_5	sy_6	sy_7	v_1	u_2/u_1	u_3/u_1	u_4/u_1	u_5/u_1	u_6/u_1	u_7/u_1
T1	0.167	0.143	0.845	0.378	0.000	0.000	128.322	0.004	0.000	0.212	0.685	0.140	0.000	1	6.4	2.3	8.3	12.4	12.4
T2	0.040	0.006	1.313	0.010	0.000	0.000	11.525	0.006	0.000	0.312	0.658	0.229	0.000	1	6.4	2.3	8.3	12.4	12.4
T3	0.261	0.240	0.560	0.579	0.000	0.000	185.682	0.002	0.000	0.129	0.623	0.095	0.000	1	6.4	2.3	8.3	12.4	12.4
T4	0.264	0.258	0.000	1.248	0.000	0.000	396.391	0.001	1.805	0.239	0.888	0.311	0.000	1	1	1	1	0.5	0.5
T5	0.071	0.037	0.732	0.447	0.000	2897.707	0.000	0.006	0.000	0.168	0.384	0.040	0.000	2	6.4	2.3	8.3	12.4	12.4
T6	0.255	0.208	0.982	0.312	0.000	0.000	118.000	0.006	0.000	0.281	0.816	0.257	0.001	1	6.4	2.3	8.3	12.4	12.4
T7	0.503	0.093	0.000	6.142	0.000	75834.349	0.000	0.075	45.394	4.977	7.750	5.565	0.000	2	1	1	1	0.5	0.5
T8	0.025	0.018	1.054	0.189	0.000	0.000	69.047	0.006	0.000	0.232	0.721	0.172	0.000	1	6.4	2.3	8.3	12.4	12.4
T9	0.198	0.117	0.000	1.499	0.000	9297.611	0.000	0.006	4.220	0.500	1.405	0.521	0.000	2	6.4	2.3	8.3	0.5	12.4
T10	0.617	0.406	1.178	0.149	0.000	0.000	57.207	0.009	0.000	0.316	0.930	0.276	0.002	1	6.4	2.3	8.3	12.4	12.4
T11	0.597	0.148	0.717	0.484	0.000	5420.246	0.000	0.007	0.000	0.198	0.335	0.005	0.001	2	6.4	2.3	8.3	12.4	12.4
T12	0.017	0.002	0.971	0.026	0.000	299.352	0.000	0.001	0.168	0.000	0.050	0.062	0.000	2	6.4	2.3	8.3	12.4	12.4
T13	1.000	0.094	1.000	0.000	0.000	0.000	0.000	0.000	0.000	0.000	0.000	0.000	0.011	2	6.4	2.3	8.3	12.4	12.4
T14	0.270	0.161	1.072	0.094	0.000	0.000	8.390	0.006	0.000	0.160	0.323	0.024	0.001	1	6.4	2.3	8.3	12.4	12.4
T15	0.112	0.010	1.213	0.003	0.000	38.670	0.000	0.006	0.000	0.208	0.554	0.111	0.001	2	6.4	2.3	8.3	12.4	12.4
T16	1.000	1.000	0.000	1.000	0.000	0.000	0.000	0.000	0.000	0.000	0.000	0.000	0.001	2	1	1	1	0.5	0.5
T18	0.062	0.052	0.898	0.326	0.000	0.000	84.876	0.007	0.000	0.227	0.431	0.081	0.000	1	6.4	2.3	8.3	12.4	12.4
T19	0.103	0.010	0.937	0.231	0.000	3155.660	0.000	0.004	0.000	0.168	0.571	0.061	0.000	2	6.4	2.3	8.3	12.4	12.4
T20	0.008	0.001	1.277	0.007	0.000	0.000	10.203	0.005	0.000	0.275	0.676	0.185	0.000	1	6.4	2.3	8.3	12.4	12.4
T21	0.003	0.000	1.205	0.000	0.000	2.953	6.642	0.003	0.000	0.199	0.566	0.089	0.000	2	6.4	2.3	8.3	12.4	12.4
T22	0.013	0.002	1.547	0.008	0.000	0.000	13.485	0.011	0.000	0.547	1.099	0.551	0.000	1	6.4	2.3	8.3	12.4	12.4
T23	0.025	0.012	1.450	0.082	0.000	0.000	23.358	0.014	0.000	0.512	1.039	0.551	0.000	1	6.4	2.3	8.3	12.4	12.4
T25	0.096	0.084	0.842	0.408	0.000	0.000	119.151	0.005	0.000	0.235	0.743	0.191	0.000	1	6.4	2.3	8.3	12.4	12.4
T26	0.115	0.014	1.363	0.107	0.000	1381.377	0.000	0.017	0.000	0.458	0.751	0.379	0.000	2	6.4	2.3	8.3	12.4	12.4

Note: DMUs T17 and T24 are excluded because their input values are zero.

S2 Table 2. Results of the DEA and DEA-AR model analyses (academia)

DMU	e	ar	L13	L16	sx_1	sy_1	sy_2	sy_3	sy_4	sy_5	sy_6	sy_7	v_1	u_2/u_1	u_3/u_1	u_4/u_1	u_5/u_1	u_6/u_1	u_7/u_1
T1	0.167	0.145	0.845	0.378	0.000	0.000	128.322	0.004	0.000	0.212	0.685	0.140	0.000	0.6	7.9	6.5	33.6	12.3	12.3
T2	0.040	0.008	1.313	0.010	0.000	0.000	11.525	0.006	0.000	0.312	0.658	0.229	0.000	0.6	7.9	6.5	33.6	12.3	12.3
T3	0.261	0.243	0.560	0.579	0.000	0.000	185.682	0.002	0.000	0.129	0.623	0.095	0.000	0.6	7.9	6.5	33.6	12.3	12.3
T4	0.264	0.260	0.000	1.248	0.000	0.000	396.391	0.001	1.805	0.239	0.888	0.311	0.000	0.6	0.8	1.2	2	0.5	0.5
T5	0.071	0.038	0.732	0.447	0.000	2897.707	0.000	0.006	0.000	0.168	0.384	0.040	0.000	2.2	7.9	6.5	33.6	12.3	12.3
T6	0.255	0.212	0.982	0.312	0.000	0.000	118.000	0.006	0.000	0.281	0.816	0.257	0.001	0.6	7.9	6.5	33.6	12.3	12.3
T7	0.503	0.095	0.000	6.142	0.000	75834.349	0.000	0.075	45.394	4.977	7.750	5.565	0.000	2.2	0.8	1.2	2	0.5	0.5
T8	0.025	0.019	1.054	0.189	0.000	0.000	69.047	0.006	0.000	0.232	0.721	0.172	0.000	0.6	7.9	6.5	33.6	12.3	12.3
T9	0.198	0.118	0.000	1.499	0.000	9297.611	0.000	0.006	4.220	0.500	1.405	0.521	0.000	2.2	7.9	6.5	33.6	0.5	12.3
T10	0.617	0.420	1.178	0.149	0.000	0.000	57.207	0.009	0.000	0.316	0.930	0.276	0.002	0.6	7.9	6.5	33.6	12.3	12.3
T11	0.597	0.154	0.717	0.484	0.000	5420.246	0.000	0.007	0.000	0.198	0.335	0.005	0.001	2.2	7.9	6.5	33.6	12.3	12.3
T12	0.017	0.003	0.971	0.026	0.000	299.352	0.000	0.001	0.168	0.000	0.050	0.062	0.000	2.2	7.9	6.5	33.6	12.3	12.3
T13	1.000	0.148	1.000	0.000	0.000	0.000	0.000	0.000	0.000	0.000	0.000	0.000	0.011	2.2	7.9	6.5	33.6	12.3	12.3
T14	0.270	0.167	1.072	0.094	0.000	0.000	8.390	0.006	0.000	0.160	0.323	0.024	0.001	0.6	7.9	6.5	33.6	12.3	12.3
T15	0.112	0.016	1.213	0.003	0.000	38.670	0.000	0.006	0.000	0.208	0.554	0.111	0.001	2.2	7.9	6.5	33.6	12.3	12.3
T16	1.000	1.000	0.000	1.000	0.000	0.000	0.000	0.000	0.000	0.000	0.000	0.000	0.001	2.2	0.8	1.2	33.6	0.5	0.5
T18	0.062	0.053	0.898	0.326	0.000	0.000	84.876	0.007	0.000	0.227	0.431	0.081	0.000	0.6	7.9	6.5	33.6	12.3	12.3
T19	0.103	0.012	0.937	0.231	0.000	3155.660	0.000	0.004	0.000	0.168	0.571	0.061	0.000	2.2	7.9	6.5	33.6	12.3	12.3
T20	0.008	0.002	1.277	0.007	0.000	0.000	10.203	0.005	0.000	0.275	0.676	0.185	0.000	0.6	7.9	6.5	33.6	12.3	12.3
T21	0.003	0.000	1.205	0.000	0.000	2.953	6.642	0.003	0.000	0.199	0.566	0.089	0.000	0.6	7.9	6.5	33.6	12.3	12.3
T22	0.013	0.002	1.547	0.008	0.000	0.000	13.485	0.011	0.000	0.547	1.099	0.551	0.000	0.6	7.9	6.5	33.6	12.3	12.3
T23	0.025	0.013	1.450	0.082	0.000	0.000	23.358	0.014	0.000	0.512	1.039	0.551	0.000	0.6	7.9	6.5	33.6	12.3	12.3
T25	0.096	0.085	0.842	0.408	0.000	0.000	119.151	0.005	0.000	0.235	0.743	0.191	0.000	0.6	7.9	6.5	33.6	12.3	12.3
T26	0.115	0.017	1.363	0.107	0.000	1381.377	0.000	0.017	0.000	0.458	0.751	0.379	0.000	2.2	7.9	6.5	33.6	12.3	12.3

Note: DMUs T17 and T24 are excluded because their input values are zero.

S2 Table 3. Results of the DEA and DEA-AR model analyses (research institutes)

DMU	e	ar	L13	L16	sx_1	sy_1	sy_2	sy_3	sy_4	sy_5	sy_6	sy_7	v_1	u_2/u_1	u_3/u_1	u_4/u_1	u_5/u_1	u_6/u_1	u_7/u_1
T1	0.167	0.144	0.845	0.378	0.000	0.000	128.322	0.004	0.000	0.212	0.685	0.140	0.000	1	7.1	7.5	19.3	13.4	13.4
T2	0.040	0.008	1.313	0.010	0.000	0.000	11.525	0.006	0.000	0.312	0.658	0.229	0.000	1	7.1	7.5	19.3	13.4	13.4
T3	0.261	0.241	0.560	0.579	0.000	0.000	185.682	0.002	0.000	0.129	0.623	0.095	0.000	1	7.1	7.5	19.3	13.4	13.4
T4	0.264	0.258	0.000	1.248	0.000	0.000	396.391	0.001	1.805	0.239	0.888	0.311	0.000	1	1	1	1	0.5	0.5
T5	0.071	0.038	0.732	0.447	0.000	2897.707	0.000	0.006	0.000	0.168	0.384	0.040	0.000	2.1	7.1	7.5	19.3	13.4	13.4
T6	0.255	0.210	0.982	0.312	0.000	0.000	118.000	0.006	0.000	0.281	0.816	0.257	0.001	1	7.1	7.5	19.3	13.4	13.4
T7	0.503	0.094	0.000	6.142	0.000	75834.349	0.000	0.075	45.394	4.977	7.750	5.565	0.000	2.1	1	1	1	0.5	0.5
T8	0.025	0.019	1.054	0.189	0.000	0.000	69.047	0.006	0.000	0.232	0.721	0.172	0.000	1	7.1	7.5	19.3	13.4	13.4
T9	0.198	0.117	0.000	1.499	0.000	9297.611	0.000	0.006	4.220	0.500	1.405	0.521	0.000	2.1	7.1	7.5	19.3	0.5	13.4
T10	0.617	0.416	1.178	0.149	0.000	0.000	57.207	0.009	0.000	0.316	0.930	0.276	0.002	1	7.1	7.5	19.3	13.4	13.4
T11	0.597	0.153	0.717	0.484	0.000	5420.246	0.000	0.007	0.000	0.198	0.335	0.005	0.001	2.1	7.1	7.5	19.3	13.4	13.4
T12	0.017	0.003	0.971	0.026	0.000	299.352	0.000	0.001	0.168	0.000	0.050	0.062	0.000	2.1	7.1	7.5	19.3	13.4	13.4
T13	1.000	0.144	1.000	0.000	0.000	0.000	0.000	0.000	0.000	0.000	0.000	0.000	0.011	2.1	7.1	7.5	19.3	13.4	13.4
T14	0.270	0.167	1.072	0.094	0.000	0.000	8.390	0.006	0.000	0.160	0.323	0.024	0.001	1	7.1	7.5	19.3	13.4	13.4
T15	0.112	0.015	1.213	0.003	0.000	38.670	0.000	0.006	0.000	0.208	0.554	0.111	0.001	2.1	7.1	7.5	19.3	13.4	13.4
T16	1.000	1.000	0.000	1.000	0.000	0.000	0.000	0.000	0.000	0.000	0.000	0.000	0.001	2.1	1	1	1	0.5	0.5
T18	0.062	0.052	0.898	0.326	0.000	0.000	84.876	0.007	0.000	0.227	0.431	0.081	0.000	1	7.1	7.5	19.3	13.4	13.4
T19	0.103	0.012	0.937	0.231	0.000	3155.660	0.000	0.004	0.000	0.168	0.571	0.061	0.000	2.1	7.1	7.5	19.3	13.4	13.4
T20	0.008	0.002	1.277	0.007	0.000	0.000	10.203	0.005	0.000	0.275	0.676	0.185	0.000	1	7.1	7.5	19.3	13.4	13.4
T21	0.003	0.000	1.205	0.000	0.000	2.953	6.642	0.003	0.000	0.199	0.566	0.089	0.000	1	7.1	7.5	19.3	13.4	13.4
T22	0.013	0.002	1.547	0.008	0.000	0.000	13.485	0.011	0.000	0.547	1.099	0.551	0.000	1	7.1	7.5	19.3	13.4	13.4
T23	0.025	0.013	1.450	0.082	0.000	0.000	23.358	0.014	0.000	0.512	1.039	0.551	0.000	1	7.1	7.5	19.3	13.4	13.4
T25	0.096	0.084	0.842	0.408	0.000	0.000	119.151	0.005	0.000	0.235	0.743	0.191	0.000	1	7.1	7.5	19.3	13.4	13.4
T26	0.115	0.017	1.363	0.107	0.000	1381.377	0.000	0.017	0.000	0.458	0.751	0.379	0.000	2.1	7.1	7.5	19.3	13.4	13.4

Note: DMUs T17 and T24 are excluded because their input values are zero.

S2 Table 4. Results of the DEA and DEA-AR model analyses (overall)

DMU	e	ar	L13	L16	sx_1	sy_1	sy_2	sy_3	sy_4	sy_5	sy_6	sy_7	v_1	u_2/u_1	u_3/u_1	u_4/u_1	u_5/u_1	u_6/u_1	u_7/u_1
T1	0.167	0.146	0.845	0.378	0.000	0.000	128.322	0.004	0.000	0.212	0.685	0.140	0.000	0.6	7.9	7.5	33.6	13.4	13.4
T2	0.040	0.009	1.313	0.010	0.000	0.000	11.525	0.006	0.000	0.312	0.658	0.229	0.000	0.6	7.9	7.5	33.6	13.4	13.4
T3	0.261	0.243	0.560	0.579	0.000	0.000	185.682	0.002	0.000	0.129	0.623	0.095	0.000	0.6	7.9	7.5	33.6	13.4	13.4
T4	0.264	0.260	0.000	1.248	0.000	0.000	396.391	0.001	1.805	0.239	0.888	0.311	0.000	0.6	0.8	1	1	0.5	0.5
T5	0.071	0.038	0.732	0.447	0.000	2897.707	0.000	0.006	0.000	0.168	0.384	0.040	0.000	2.2	7.9	7.5	33.6	13.4	13.4
T6	0.255	0.213	0.982	0.312	0.000	0.000	118.000	0.006	0.000	0.281	0.816	0.257	0.001	0.6	7.9	7.5	33.6	13.4	13.4
T7	0.503	0.095	0.000	6.142	0.000	75834.349	0.000	0.075	45.394	4.977	7.750	5.565	0.000	2.2	0.8	1	1	0.5	0.5
T8	0.025	0.019	1.054	0.189	0.000	0.000	69.047	0.006	0.000	0.232	0.721	0.172	0.000	0.6	7.9	7.5	33.6	13.4	13.4
T9	0.198	0.118	0.000	1.499	0.000	9297.611	0.000	0.006	4.220	0.500	1.405	0.521	0.000	2.2	7.9	7.5	33.6	0.5	13.4
T10	0.617	0.422	1.178	0.149	0.000	0.000	57.207	0.009	0.000	0.316	0.930	0.276	0.002	0.6	7.9	7.5	33.6	13.4	13.4
T11	0.597	0.155	0.717	0.484	0.000	5420.246	0.000	0.007	0.000	0.198	0.335	0.005	0.001	2.2	7.9	7.5	33.6	13.4	13.4
T12	0.017	0.003	0.971	0.026	0.000	299.352	0.000	0.001	0.168	0.000	0.050	0.062	0.000	2.2	7.9	7.5	33.6	13.4	13.4
T13	1.000	0.158	1.000	0.000	0.000	0.000	0.000	0.000	0.000	0.000	0.000	0.000	0.011	2.2	7.9	7.5	33.6	13.4	13.4
T14	0.270	0.168	1.072	0.094	0.000	0.000	8.390	0.006	0.000	0.160	0.323	0.024	0.001	0.6	7.9	7.5	33.6	13.4	13.4
T15	0.112	0.017	1.213	0.003	0.000	38.670	0.000	0.006	0.000	0.208	0.554	0.111	0.001	2.2	7.9	7.5	33.6	13.4	13.4
T16	1.000	1.000	0.000	1.000	0.000	0.000	0.000	0.000	0.000	0.000	0.000	0.000	0.001	2.2	0.8	1	1	0.5	0.5
T18	0.062	0.053	0.898	0.326	0.000	0.000	84.876	0.007	0.000	0.227	0.431	0.081	0.000	0.6	7.9	7.5	33.6	13.4	13.4
T19	0.103	0.012	0.937	0.231	0.000	3155.660	0.000	0.004	0.000	0.168	0.571	0.061	0.000	2.2	7.9	7.5	33.6	13.4	13.4
T20	0.008	0.002	1.277	0.007	0.000	0.000	10.203	0.005	0.000	0.275	0.676	0.185	0.000	0.6	7.9	7.5	33.6	13.4	13.4
T21	0.003	0.000	1.205	0.000	0.000	2.953	6.642	0.003	0.000	0.199	0.566	0.089	0.000	0.6	7.9	7.5	33.6	13.4	13.4
T22	0.013	0.002	1.547	0.008	0.000	0.000	13.485	0.011	0.000	0.547	1.099	0.551	0.000	0.6	7.9	7.5	33.6	13.4	13.4
T23	0.025	0.013	1.450	0.082	0.000	0.000	23.358	0.014	0.000	0.512	1.039	0.551	0.000	0.6	7.9	7.5	33.6	13.4	13.4
T25	0.096	0.085	0.842	0.408	0.000	0.000	119.151	0.005	0.000	0.235	0.743	0.191	0.000	0.6	7.9	7.5	33.6	13.4	13.4
T26	0.115	0.017	1.363	0.107	0.000	1381.377	0.000	0.017	0.000	0.458	0.751	0.379	0.000	2.2	7.9	7.5	33.6	13.4	13.4

Note: DMUs T17 and T24 are excluded because their input values are zero.
